# Rationale and design of the Clinical Evaluation of Magnetic Resonance Imaging in Coronary heart disease 2 trial (CE-MARC 2): A prospective, multicenter, randomized trial of diagnostic strategies in suspected coronary heart disease

**DOI:** 10.1016/j.ahj.2014.10.008

**Published:** 2015-01

**Authors:** David P. Ripley, Julia M. Brown, Colin C. Everett, Petra Bijsterveld, Simon Walker, Mark Sculpher, Gerry P. McCann, Colin Berry, Sven Plein, John P. Greenwood

**Affiliations:** a Multidisciplinary Cardiovascular Research Centre (MCRC) & Leeds Institute of Cardiovascular and Metabolic Medicine, University of Leeds, Leeds, UK; b Leeds Institute of Clinical Trials Research, University of Leeds, Leeds, UK; c Centre for Health Economics, University of York, York, UK; d Department of Cardiovascular Sciences, University of Leicester, and the National Institute of Health Research Leicester Cardiovascular Biomedical Research Unit, Leicester, UK; e BHF Glasgow Cardiovascular Research Centre, Institute of Cardiovascular and Medical Sciences, University of Glasgow, Glasgow, UK

## Abstract

**Background:**

A number of investigative strategies exist for the diagnosis of coronary heart disease (CHD). Despite the widespread availability of noninvasive imaging, invasive angiography is commonly used early in the diagnostic pathway. Consequently, approximately 60% of angiograms reveal no evidence of obstructive coronary disease. Reducing unnecessary angiography has potential financial savings and avoids exposing the patient to unnecessary risk. There are no large-scale comparative effectiveness trials of the different diagnostic strategies recommended in international guidelines and none that have evaluated the safety and efficacy of cardiovascular magnetic resonance.

**Trial Design:**

CE-MARC 2 is a prospective, multicenter, 3-arm parallel group, randomized controlled trial of patients with suspected CHD (pretest likelihood 10%-90%) requiring further investigation. A total of 1,200 patients will be randomized on a 2:2:1 basis to receive 3.0-T cardiovascular magnetic resonance–guided care, single-photon emission computed tomography–guided care (according to American College of Cardiology/American Heart Association appropriate-use criteria), or National Institute for Health and Care Excellence guidelines–based management. The primary (efficacy) end point is the occurrence of unnecessary angiography as defined by a normal (>0.8) invasive fractional flow reserve. Safety of each strategy will be assessed by 3-year major adverse cardiovascular event rates. Cost-effectiveness and health-related quality-of-life measures will be performed.

**Conclusions:**

The CE-MARC 2 trial will provide comparative efficacy and safety evidence for 3 different strategies of investigating patients with suspected CHD, with the intension of reducing unnecessary invasive angiography rates. Evaluation of these management strategies has the potential to improve patient care, health-related quality of life, and the cost-effectiveness of CHD investigation.

## Background

Coronary heart disease (CHD) is a leading cause of death and disability worldwide. In a typical hospital setting, a variety of investigations may be used to diagnose CHD, risk-stratify, and determine the need for coronary revascularization. Myocardial perfusion scintigraphy by single-photon emission computed tomography (SPECT) is the most commonly used test worldwide for the assessment of myocardial ischemia, and there is a large body of evidence to support its prognostic value. Cardiovascular magnetic resonance (CMR) has high diagnostic accuracy for the detection of CHD, and the CE-MARC study demonstrated CMR's superiority over SPECT.^1^ Despite the widespread availability and recommendation of noninvasive imaging investigations in national and international guidelines,^2–4^ invasive coronary angiography is commonly used early in the diagnostic pathway. Evidence from large populations of patients presenting with chest pain has confirmed that the majority will not have significant obstructive coronary disease.^5,6^ In the United States, the American College of Cardiology National Cardiovascular Data Registry identified almost 400,000 patients without known CHD that underwent elective catheterization from January 2004 through April 2008, and only 38% had obstructive CHD.^6^

Avoiding unnecessary angiography has potential financial savings and avoids exposing the patient to unnecessary risk. Invasive coronary angiography has a risk of major complications of 1.7%. Furthermore, the dose and stochastic effects of x-ray radiation are frequently misjudged,^7^ with the risk of developing a solid tumor estimated at 1:2500 diagnostic coronary angiographic procedures.^8^ Paradoxically, the implementation of UK national guidelines for the assessment and diagnosis of recent onset chest pain has been demonstrated to increase invasive coronary angiography rates between 20% and 28%.^9^

A previous single-center trial (CECaT) indicated that invasive angiography could be avoided in 20% to 25% of patients using functional testing as an initial gatekeeper.^10^ To date, there are no large-scale comparative effectiveness trials of the different diagnostic strategies recommended in international guidelines and none that have evaluated the safety and efficacy of CMR.

## Study objectives

The primary objectives are to determine if 3.0-T CMR-guided management is superior to (*a*) National Institute for Health and Care Excellence (NICE) guidelines–based management (CG95),^3^ and (*b*) SPECT-guided management,^11^ in terms of reducing the rates of unnecessary invasive angiography occurring within 12 months in patients with a pretest likelihood (PTL) of CHD of 10% to 90%.

Secondary objectives will determine (*a*) if in patients with a high PTL of CHD (61%-90%), noninvasive imaging (CMR or SPECT) is superior to NICE guidelines–based management, in terms of reducing the occurrence of unnecessary invasive angiography; (*b*) safety in terms of major adverse cardiovascular events (MACE) at 3 years between the CMR-guided care group and those receiving NICE guidelines–based management; (*c*) safety in terms of MACE at 3 years between the CMR-guided care group and those receiving SPECT-guided management; and (*d*) cost-effectiveness and impact on health-related quality-of-life (HRQoL) measures of a CMR-guided care strategy compared with NICE guidelines–based management and SPECT-guided management.

## Methods

### Study design

**Figure 1:**
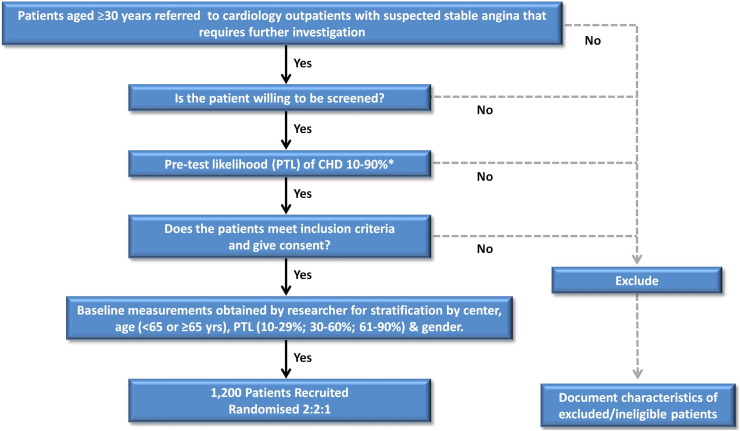
CE-MARC 2 recruitment process. *PTL as defined by NICE (CG95) guidelines.^3^

**Figure 2:**
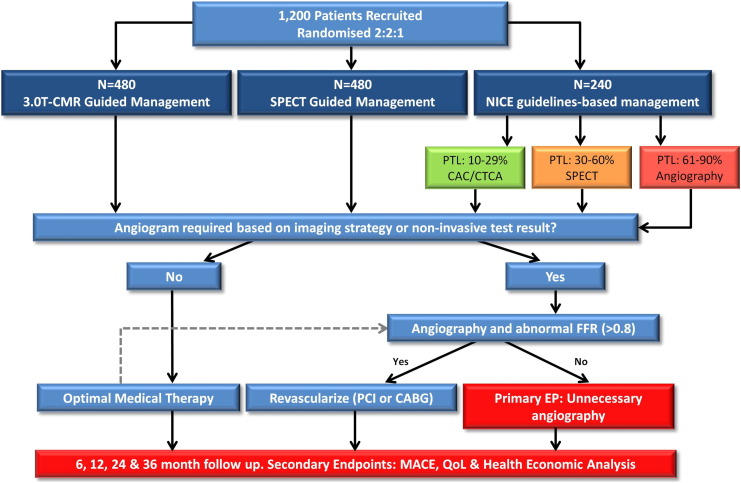
CE-MARC 2 study flow diagram.

CE-MARC 2 (clinicaltrials.gov: NCT01664858) is a prospective, multicenter, multivendor, 3-arm parallel group, randomized controlled trial of patients referred to cardiology care for further evaluation of symptoms thought to be angina pectoris. A total of 1,200 patients with suspected CHD will be randomized on a 2:2:1 basis to receive CMR-guided care, SPECT-guided care, or NICE guidelines–based management (Figures 1 and 2).

Statistical analysis will be performed by the Clinical Trials Research Unit, University of Leeds, and the Centre for Health Economics, University of York. The study population will be followed up prospectively for a minimum of 3 years to establish long-term MACE in each investigation arm. The study will be conducted in accordance with the Declaration of Helsinki and has been approved by the National Research Ethics Service.

### Patient Population, Recruitment, and Randomization

Subjects will be considered for inclusion if they are 30 years or older presenting to participating hospitals (Appendix A) with suspected cardiac chest pain (angina) with a defined CHD PTL of 10% to 90%.^3^ Full inclusion and exclusion criteria are listed in Table I. An anonymized log of all patients screened for eligibility who are not recruited either because they are ineligible or because they declined to participate will be kept.

The treating clinician makes a clinical diagnosis of typical angina if the patient has all 3 salient features of angina (constricting discomfort in the front of the chest, or in the neck, shoulders, jaw, or arms; precipitated by physical exertion; and relieved by rest or glyceryl trinitrate within ~5 minutes) or atypical angina if they have 2 of 3.^3,12^ Those with one or none of the features are defined as nonanginal chest pain^3,12^ and ineligible for the study. The patients' risk factors (age, gender, ethnicity, abdominal and hip circumference, lipid profile, blood pressure, smoking, and diabetic status), medical history (including rheumatoid arthritis, hypertension, hyperlipidemia, peripheral vascular disease, and cerebrovascular disease), and family history of premature CHD will be recorded.

Patients will undergo risk stratification with their PTL of having CHD calculated^3,13^ and categorized as low (10%-29%), intermediate (30%-60%), or high (61%-90%). Randomization will be achieved using minimization, incorporating a random element through a computer-generated program accessed via a 24-hour telephone service. This will allocate patients in a 2:2:1 ratio between CMR/SPECT/NICE after taking account of the following stratification factors: randomizing site, age (30-64 years, ≥65 years), PTL (10%-29%, 30%-60%, 61%-90%), and gender. Those with low PTL of underlying CHD (10%-29%) randomized to NICE guidelines will undergo cardiac computed tomography (CCT), intermediate PTL (30%-60%) SPECT, and high PTL (61%-90%) coronary angiography.

## Funding

The trial was funded by the British Heart Foundation (SP/12/1/29062). Additional support was received from the Leeds Teaching Hospital Charitable Foundation and the National Institute for Health Research, through the Local Clinical Research Networks.

The authors are solely responsible for the design and conduct of this study, all study analyses, the drafting and editing of the manuscript, and its final contents.

### Investigation Details

#### Cardiovascular magnetic resonance

Cardiovascular magnetic resonance will be performed on a clinical 3.0-T scanner using protocols that conform to international standards.^14^ A cardiac imaging receiver coil configuration will be used, and electrocardiogram (ECG) gating will be performed. The scan will comprise the following:

1. Survey and reference scans prior to defining the short, vertical long, and horizontal long axes acquired with a balanced steady-state free precession (bSSFP), single-slice breath-hold sequence. bSSFP pulse sequence parameters dependent on scanner manufacturer and site. Typical parameters are as follows: echo time 1.3 milliseconds, repetition time 2.6 milliseconds, flip angle 40°, field of view 320-420 mm according to patient size, SENSE or GRAPPA acceleration, slice thickness 10 mm, and 30 phases per cardiac cycle.
2. Stress perfusion imaging performed with adenosine administered initially at 140 μg kg^−1^ min^−1^. Adequate hemodynamic response is assessed by either ≥10% heart rate increase or ≥10 mm Hg decrease in systolic blood pressure. If there is inadequate hemodynamic response, then the dose will be increased incrementally to 170 μg kg^−1^ min^−1^ and then 210 μg kg^−1^ min^−1^ for a further 2 minutes until hemodynamic response is achieved.
  Perfusion image acquisition will use a 2-dimensional, *T*_1_-weighted saturation recovery–prepared gradient echo-pulse sequence in 3 short-axis slices, planned using the 3/5 technique,^15^ using either parallel imaging acceleration (SENSE or GRAPPA) or spatiotemporal undersampling (5× *kt*-BLAST). First-pass contrast-enhanced study will be performed using a dual-bolus technique (0.075 mmol/kg gadobutrol [Gadovist; Bayer Schering Pharma, Berlin, Germany]) for the main bolus preceded by the same volume of a 10% dilute contrast agent dose for the prebolus, both administered at a rate of 4.0 mL/s followed by a 20-mL saline flush.
3. Resting wall motion and left ventricular function will be assessed with a contiguous stack of multiphase ventricular short-axis bSSFP cines (10-12 slices, 30 phases, 10-mm slice thickness, 0-mm gap, same cine pulse sequence as above).
4. The rest myocardial perfusion study will use identical pulse sequence, slice positioning, and injection characteristics to the stress perfusion scan. If the stress perfusion scan was not of adequate quality (eg, ectopics and failure to trigger), a repeat stress may be performed as alternative to the rest study.
5. Late gadolinium enhancement (LGE) performed in 10 to 12 short-axis slices 10 to 15 minutes after step 4 with an inversion recovery–prepared *T*_1_-weighted gradient echo-pulse sequence. Typical parameters are as follows: echo time 2.0 milliseconds, repetition time 3.7 milliseconds, flip angle 25°, acquired spatial resolution 0.70 × 0.70 × 10 mm^3^, and inversion time individually adjusted according to inversion time scout. LGE will be acquired with alternate heart beat acquisition (with single-shot or navigated LGE, an option for poor breath holders) and long-axis and modified views acquired if clinically indicated.

#### Single-photon emission computed tomography

Radionuclide imaging will be performed according to local standard departmental practice conforming to both national and international guidelines.^16–18^ Patients will undergo either a 1- or 2-day scanning protocol with a radioisotope tracer ^99m^Tc-tetrofosmin or ^99m^Tc-sestamibi (Myoview, GE Healthcare and CARDIOLITE, Lantheus Medical Imaging). A weight-adjusted dose up to a maximum of 1000 MBq per examination will be used for stress and rest imaging, which will be performed within 5 days of each other.

Stress examination will be performed with either treadmill or bicycle exercise, pharmacologic vasodilator stress (with adenosine or regadenoson), or a combination. Treadmill will involve exercise using the BRUCE or modified BRUCE protocol or bicycle ergometer typically commencing at 25 W increasing workload by 25 W every 2 minutes. Radioisotope tracer will be injected at peak stress.

If pharmacologic stress with adenosine is used, the administration regimen will be comparable with the CMR protocol. If regadenoson is used, 0.4 mg will be delivered by rapid intravenous injection. Radioisotope tracer will be injected after at least 4 minutes of adequate hemodynamic/symptom response. Vasodilator stress may be combined with submaximal exercise.

Images will be acquired on either a dual headed gamma camera or solid-state cadmium zinc telluride camera. Stress and rest images will be gated to the ECG, and attenuation correction will be used where routinely available.

#### Cardiac CT

Cardiac computed tomography will be performed on a minimum 64-slice multidetector CT and follow international guidelines.^19^ Coronary artery calcium (CAC) scoring scan protocol will involve the following:

1. Scout scans.
2. Unenhanced scan with prospective gating and inspiratory breath-hold. A minimum scan length (*z*-axis distance) will be used from tracheal bifurcation to the inferior border of the heart.
3. Agatson CAC score will be calculated and NICE guidance followed.^3^ If CAC is 0, no further imaging will be performed; if CAC score is 1 to 400, proceed to CT coronary angiography (CTCA); and if CAC score is >400, refer for invasive coronary angiography.

For CT coronary angiography, heart rate control will be achieved with β-blockade (intravenous or oral) and short-acting sublingual nitrates given. Computed tomography coronary angiography will be performed where possible, with a prospective gating technique using the minimum scan range planned from the unenhanced scan. Typical scan parameters are as follows: 0.625-mm collimation, pitch 0.2-0.4, 100-120 kV, and 400-830 mAs (adjusted according to body mass index). If retrospective gating is required, ECG dose modulation will be used to minimize radiation dose. The acquisition window will usually be centered at end-diastole (end-systole may be used at the discretion of the attending physician). In those with variable heart rates, time interval padding may be used to allow reconstruction of both the systolic and diastolic phase data sets. The exact scan parameters and radiation reduction algorithms used will be dependent on the hardware vendor. Iodinated contrast agent of 60 to 120 mL will be administered at a flow rate of 4.5 to 6.5 mL/s, followed by a bolus of normal saline (eg, 50 mL and 5 mL/s) during an acquisition with inspiratory breath-hold. Either a test bolus or a bolus tracking technique may be used.

#### Invasive angiography and Fractional Flow Reserve

Angiography will be performed using a standard technique (radial or femoral approach). Fractional flow reserve (FFR) (PressureWire; St Jude Medical, Minneapolis, MN) will be performed in all vessels ≥2.5 mm with stenosis ≥40% and ≤90%, following intracoronary nitrates, using adenosine at 140 to 210 μg kg^−1^ min^−1^ to achieve maximal hyperemia and hemodynamic steady state; pull-back assessment of diffuse disease or serial stenoses can be made. Adenosine will be administered as per CMR protocol. Totally occluded coronary arteries will have a default FFR value of 0.50 recorded; for lesions >90%, FFR will also be considered positive (0.50), and for lesions <40%, FFR will be considered normal (0.90).^20^ All sites will have FFR quality assurance core laboratory assessment of the FFR recordings using vendor software (RADIVIEW 2.2; St Jude Medical Corp).

### Investigation reporting

All test results will be reported by independent cardiology/radiology consultants with a minimum 5-year experience in the imaging modality. In accordance with usual clinical practice, all clinical data from all noninvasive imaging modalities will be available for the reporting physician to make an overall clinical judgement.

#### Cardiovascular magnetic resonance

Cardiovascular magnetic resonance analysis will be both visual and quantitative following international recommendations.^21^ Local on-site reporting will include regional wall motion abnormalities (by visual analysis using the 17-segment American Heart Association/American College of Cardiology model). Each segment scored as 0 = normal, 1 = mild hypokinesia, 2 = severe hypokinesia, 3 = akinesia, or 4 = dyskinesia. Quantitative analysis will include the following: end-diastolic volume (mL), end-systolic volume (mL), stroke volume (mL), and ejection fraction (%).

Detection of hypoperfusion (ischemia), by visual comparison of stress, rest, and LGE scans, will be scored as 0 = normal, 1 = equivocal, 2 = nontransmural ischemia <50%, 3 = nontransmural ischemia ≥50%, or 4 = transmural ischemia in 16 segments of the 17-segment American Heart Association/American College of Cardiology model (excluding the apical cap).

Any infarct (scar) will be reported based on the LGE images (17-segment model), with scores of 0 = no hyperenhancement, 1 = 1%-25% mural thickness, 2 = 26%-50%, 3 = 51%-75%, or 4 = >75% allocated to each segment.

A positive result (≥2 adjacent segments [or 60° arc-equivalent if the defect crosses segmental boundaries] with ≥50% transmural extent of ischemia, scar, or ischemia-scar combination) will by protocol necessitate referral for invasive angiography ± FFR

#### Single-photon emission computed tomography

Single-photon emission computed tomography analysis will be both visual and quantitative. Local on-site reporting will include any regional wall motion abnormality (by visual analysis using the 17-segment model). Each segment scored as 0 = normal, 1 = mild hypokinesia, 2 = severe hypokinesia, 3 = akinesia, or 4 = dyskinesia.

Evidence of ischemia, by visual comparison of rest and stress scans, will be scored as 0 = normal, 1 = mild 51%-70%, 2 = moderate 31%-50%, 3 = severe 10%-30%, or 4 = absent <10% in each segment. Quantitative analysis will include summed rest score and summed stress scores, quantitative perfusion SPECT (QPS) defect extent (%), quantitative perfusion SPECT total perfusion deficit (%), end-systolic volume (mL), end-diastolic volume (mL), stroke volume (mL), and ejection fraction (%).

The presence of artifacts including subdiaphragmatic activity affecting the inferior wall, significant patient movement, anterior attenuation, inferior attenuation, and left bundle-brunch block artifact will be recorded.

A positive result (summed stress score ≥ 4), unless believed by the reporting clinician to represent attenuation artifact, will by protocol necessitate referral for invasive angiography ± FFR.

#### Cardiac CT

The total Agatson CAC score from the unenhanced scan will be determined. If the CAC score is >0 and <400, a contrast-enhanced scan will be performed.

Coronary stenosis will be graded as 0 = normal, 1 = minimal <25% stenosis, 2 = mild 25%-49%, 3 = moderate 50%-69%, 4 = severe 70%-99%, or 5 = occluded 100%. A positive result (either CAC >400 or any luminal stenosis ≥50% in an epicardial coronary artery ≥2.5-mm diameter) will by protocol necessitate invasive angiography ± FFR.

#### X-ray angiography and FFR

Invasive x-ray angiography will be interpreted visually by the performing clinician recording the coronary artery dominance, location, and visual degree (%) of all coronary stenoses in all major epicardial coronary arteries (with luminal diameter ≥2.5 mm). FFR measurement will be recorded in all arteries ≥2.5 mm with a visually recorded diameter stenosis ≥40% and ≤90%. Where FFR cannot be performed due to clinical/safety reasons, quantitative coronary angiography (QCA) will be performed using validated commercial vendor software. In this instance, QCA measurements will be made during offline analysis by a single independent blinded observer at the Glasgow Angiographic core laboratory. Lesions will be considered significant if a coronary artery segment (luminal diameter ≥2.5 mm) analyzed by QCA has a percentage diameter stenosis of ≥70% in one view or ≥50% in 2 orthogonal views.

### Protocol deviations

On occasion where the attending cardiologist overrules the protocol requirement to proceed to invasive coronary angiography, this will be recorded as a protocol violation.

### Annual follow-up

Annual follow-up over the subsequent 3 years will be undertaken to record death (including cause), other MACE, and withdrawal. For alive patients, medical history since randomization, including details and dates of acute coronary syndrome (ACS), emergency or elective revascularization procedure, any admission for cardiovascular cause will be obtained and verified from hospital or family practitioner records. Details of any recent cardiovascular investigations will be taken. In addition, Office for National Statistics monitoring will be sought for deceased patients to determine the certified causes of death.

### Primary end point

The primary end point is unnecessary invasive coronary angiography occurring within 12 months in each arm. This will be defined at the time of coronary angiography by an FFR measurement of >0.80 in all vessels ≥2.5 mm in a patient-based analysis (ie, at least 1 vessel with an FFR measurement of <0.8 will be required to define a patient with disease).

An “unnecessary angiogram” will be defined as one of the following:

• A negative FFR and positive noninvasive test result (ie, a false-positive test result)
• A negative FFR in a high PTL (61%-90%) patient that proceeds directly to invasive angiography in the NICE guidelines–based strategy arm (ie, a false-positive for the strategy)
• A negative FFR and a negative noninvasive test result (ie, a true-negative strategy result in which the imaging result was “not believed” by the treating cardiologist—based on intention-to-treat principles)
• A negative FFR and an inconclusive noninvasive test result in which angiography had to be performed to make the diagnosis (ie, failure of the strategy to produce a diagnosis)

### Secondary end points

#### Major adverse cardiovascular events

For all patients, MACE at 12 months and a minimum of 3 years will be reported. Major adverse cardiovascular events will be defined as death due to cardiovascular cause, myocardial infarction (MI; defined by the Third Universal Definition^22^), unplanned coronary revascularization, and hospital admission for cardiovascular cause. Hospitalization for cardiovascular cause will be defined as troponin-negative ACS, spontaneous MI (type 1), MI secondary to ischemic imbalance (type 2), MI related to stent thrombosis (type 4b), arrhythmia, stroke, and heart failure.

#### Positive coronary angiogram

The proportion of patients in the relevant population who undergo an invasive coronary angiogram yielding a positive finding by FFR within 12 months of randomization will be determined.

#### Economic evaluation

To assess the long-term cost-effectiveness of the alternate diagnostic testing strategies, information from the trial will be used to update the economic model developed as part of the CE-MARC trial.^23^ The model will use information from the trial, including resource use, costs, HRQoL, and other clinical outcomes (eg, on unnecessary tests and MACE events), together with epidemiologic, clinical, and economic data from other sources to calculate costs and quality-adjusted life-years for patients. The economic evaluation will use methods consistent with those recommended by NICE.^24^ Given the potential difference between diagnostic strategies in terms of mortality, the modeling will adopt a lifetime time horizon to capture any difference.

#### Quality of life

Health-related quality of life will be measured by the following:

- Seattle Angina Questionnaire–UK version
- Medical Outcomes Survey–Short Form 12
- EuroQol 5-Dimensions.

#### Complications

Complications directly related to investigational or procedural aspects of the study resulting in prolonged hospital stay/specific treatment that would otherwise have not been required will be reported. These will be established and adjudicated by the Trial Steering Committee and Trial Management Group and reported to the Data Monitoring and Ethics Committee (DMEC).

### Statistical considerations

#### Sample size

Sample size calculations were performed using nQuery 7.0, Statistical Solutions Ltd., Cork, Ireland. For the primary end point analyses, a sample size of 1,200 (allowing for 20% noncompletion) will provide 99% power to detect a difference of unnecessary angiography rates between CMR and NICE guidelines–based management—accounting for the 2:1 allocation ratio—and 94% power between CMR and SPECT-guided care (2-sided test 5% significance level for a continuity-corrected χ^2^ test^24^). This is based on projected unnecessary angiography rates of 4.5%, 11.7%, and 30% in the CMR, SPECT, and NICE arms, respectively, arrived at by estimating the PTL profile of CEMARC patients (we estimated the PTL distribution to be 10%:33%:57% for low/moderate/high PTL, for those patients with PTL 10%-90%) and the false-positive rates of CMR and SPECT in these subgroups to compute a weighted average false-positive rate as the expected unnecessary angiogram rate. For the NICE arm, we noted that in patients with 61% to 90% PTL, nearly 60% of angiograms were negative, and so would drive high rates for this strategy, despite CT and SPECT patients (10%-60% PTL) undergoing fewer unnecessary angiograms.

#### Analysis plan

Statistical analysis will be performed as agreed in the prespecified statistical analysis plan. All analyses will be performed on intention-to-treat basis. The primary end point will be performed after the 12-month assessment has been completed by the last patient entered into the study and a complete and exhaustive data chase has been performed. Analyses of primary and secondary end points will be performed separately for the CMR-guided vs NICE-guided care, CMR-guided vs SPECT-guided care, and SPECT-guided vs NICE-guided care comparisons.

#### Primary end point analysis

The difference in proportions of patients randomized to each arm with a study-defined unnecessary angiogram and 95%CI for this difference will be presented for summary purposes. A binary logistic regression will model the relative odds of receiving an unnecessary angiogram for CMR-guided care vs the group of interest (either NICE or SPECT-guided care pathways) when controlling for the minimization factors. The estimated odds ratios, 95% CI, and *P* values will be presented. An unadjusted analysis will compare the difference in the proportions between the 2 groups using a χ^2^ test.

### Secondary end point analysis

#### Major adverse cardiovascular events

The proportions of patients in the 3 groups with a MACE at 12 and 36 months and absolute differences in these MACE rates will be presented. This analysis will be performed both on the intention-to-treat and per-protocol basis. Periprocedural MI—type 4a (related to percutaneous coronary intervention [PCI]) and type 5 (related to coronary artery bypass grafting)—and planned revascularization (PCI or coronary artery bypass grafting) based on the index FFR results will be censored.

#### Quality of life

The scores will be presented for the groups at 6, 12, 24, and 36 months. The scores for the dimensions of the Seattle Angina Questionnaire and Medical Outcomes Survey-Short Form 12 will be summarized by randomized group at each time point. Multilevel repeated-measures modeling will be used to estimate differences between the groups at all postbaseline time points (allowing for time, trial group, and trial group by time interaction, and adjusting for baseline QoL and minimization factors [all fixed effects], and for patient and patient by time interaction [random effects]). Residuals and predicted values produced from the multivariate models will be examined to assess the assumptions of the statistical model.

### Data monitoring

Data will be monitored for completeness and quality by the Clinical Trials Research Unit. A full monitoring schedule including serious adverse events and adverse reactions will be established and agreed by the Trial Steering Committee and Trial Management Group. Ethical and safety considerations will be monitored by the DMEC (Appendix B). A quality assurance process will be undertaken centrally by independent modality-specific imaging experts, to monitor the quality of image acquisition and interpretation of each imaging modality at all recruiting centers. This will involve an initial review of the first 15 imaging studies followed by an ongoing review of a random 10% of each imaging modality at each participating site. Clinical interpretation of the individual components of each imaging modality and overall study recommendation will be scored as 1 (agreement), 2 (minor disagreement), or 3 (major disagreement) and reported to the DMEC for independent consideration/action.

## Conclusion

The CE-MARC 2 trial is a prospective, multicenter, 3-arm parallel group, randomized controlled trial; it will provide comparative efficacy and safety evidence for 3 different strategies of investigating patients with suspected CHD, with the intention of reducing unnecessary invasive angiography rates. Evaluation of these management strategies has the potential to improve patient care, HRQoL, and the cost-effectiveness of CHD investigation.

## Disclosures

S.P. is funded by a British Heart Foundation fellowship (FS/10/62/28409). C.B. has had educational and research grants and acted as a consultant for St Jude Medical Corporation with institutional reimbursement to the University of Glasgow. G.P.M. is funded by a National Institute of Health Research fellowship.

## Figures and Tables

**Table I:** Inclusion and exclusion criteria into the CE-MARC2 study

Inclusion criteria	Exclusion eriteria
Age ≥30 y	Nonanginal chest pain
Suspected stable angina (CHD) that requires further investigation	Normal SPECT/CCT within the last 2 y
A defined PTL of 10%-90%	Clinically unstable
Suitable for revascularization if required	Previous MI or biomarker positive ACS
	Previous revascularization with coronary artery bypass surgery or PCI
	Contraindication to CMR imaging
	known adverse reaction to adenosine or gadolinium/iodinated contrast agents
	Obesity (where body girth exceeds scanner diameter)
	Pregnancy and/or breast-feeding
	Known chronic renal failure (estimated glomerular filtration rate <30 mL/min per 1.73 m^2^)
	Inability to give informed consent

## Appendix A Recruitment Centers

Leeds General Infirmary, Leeds, UK (CI: Prof. J.P. Greenwood, local PI: Prof. S. Plein).

Glenfield Hospital, Leicester, UK (PI: Dr G.P. McCann).

Greater Glasgow and Clyde Hospitals, UK (PI: Prof. C. Berry).

John Radcliffe Hospital, Oxford, UK (PI: Dr E. Dall'Armellina).

Bristol Royal Infirmary, Bristol, UK (PI: Dr C. Bucciarelli-Ducci).

St George's Hospital, London, UK (PI: Prof. A. Prasad).

## Appendix B Trial management structure

**Trial Management Group:** Prof. J.P. Greenwood (Chief Investigator [Chair]), Prof. S. Plein (Co-investigator), Prof. C. Berry (Co-investigator), Dr G.P. McCann (Co-investigator), Prof. Mark Sculpher (Co-investigator), Dr D.P. Ripley (Clinical Research Fellow), Ms P. Bijsterveld (Senior Research Nurse), Dr M. Bhogal (Senior Trial Co-ordinator), Ms C. Reynolds (Data Manager), Prof. J. Brown (Supervising Statistician), Mr C. Everett (Statistician), and Ms S. Hartley (Trial Manager).

**Trial Steering Committee:** Independent members comprise Professor A.L. Clark (Chair), Prof. A. Kelion (SPECT expert), Dr C. Peebles (CCT expert), Dr S. Wheatcroft (Interventionist), Dr M. Kelly (Independent Statistician), and Mr G. Oliver (patient support group representative). In addition, members of the Trial Management Group include Prof. J.P. Greenwood, Dr D.P. Ripley, Ms P. Bijsterveld, Dr M. Bhogal, Ms C. Reynolds, Prof. J. Brown, Mr C. Everett, and Ms S. Hartley.

**Data Monitoring and Ethics Committee:** Professor J.J. Deeks (Independent Chair), Dr J. Gunn (Independent member), Dr N. Maredia (Independent member), and Dr D. Blackman (Independent member).
